# The Impact of Personal Background and School Contextual Factors on Academic Competence and Mental Health Functioning across the Primary-Secondary School Transition

**DOI:** 10.1371/journal.pone.0089874

**Published:** 2014-03-07

**Authors:** Sharmila Vaz, Richard Parsons, Torbjörn Falkmer, Anne Elizabeth Passmore, Marita Falkmer

**Affiliations:** 1 School of Occupational Therapy and Social Work, Centre for Research into Disability and Society, Curtin Health Innovation Research Institute, Curtin University, Perth, Western Australia, Australia; 2 School of Pharmacy, Curtin Health Innovation Research Institute, Curtin University, Perth, Western Australia, Australia; 3 School of Occupational Therapy and Social Work, Curtin Health Innovation Research Institute, Curtin University, Perth, Western Australia, Australia; 4 School of Occupational Therapy, La Trobe University, Melbourne, Victoria, Australia; 5 Rehabilitation Medicine, Department of Medicine and Health Sciences (IMH), Faculty of Health Sciences, Linköping University & Pain and Rehabilitation Centre, UHL, County Council, Linköping, Sweden; 6 School of Education and Communication, CHILD programme, Institution of Disability Research Jönköping University, Jönköping, Sweden; University of Bath, United Kingdom

## Abstract

Students negotiate the transition to secondary school in different ways. While some thrive on the opportunity, others are challenged. A prospective longitudinal design was used to determine the contribution of personal background and school contextual factors on academic competence (AC) and mental health functioning (MHF) of 266 students, 6-months before and after the transition to secondary school. Data from 197 typically developing students and 69 students with a disability were analysed using hierarchical linear regression modelling. Both in primary and secondary school, students with a disability and from socially disadvantaged backgrounds gained poorer scores for AC and MHF than their typically developing and more affluent counterparts. Students who attended independent and mid-range sized primary schools had the highest concurrent AC. Those from independent primary schools had the lowest MHF. The primary school organisational model significantly influenced post-transition AC scores; with students from Kindergarten - Year 7 schools reporting the lowest scores, while those from the Kindergarten - Year 12 structure without middle school having the highest scores. Attending a school which used the Kindergarten - Year 12 with middle school structure was associated with a reduction in AC scores across the transition. Personal background factors accounted for the majority of the variability in post-transition AC and MHF. The contribution of school contextual factors was relatively minor. There is a potential opportunity for schools to provide support to disadvantaged students before the transition to secondary school, as they continue to be at a disadvantage after the transition.

## Introduction

### The issue: Transition from primary to secondary school

The transition from primary to secondary school has long been acknowledged as an important change in the lives of most students [1]–[3]. Despite contextual variations in school systems, similarities in the features of this transition exist [4]. Typically, the secondary school transition involves simultaneous changes in school environments, relationships, and academic expectations [1], [5]–[7]. Students in Western Societies, including Australia, negotiate the school transition at a time in development when they are striving to gain independence from their parents, establish a unique identity [8], [9], and gain approval and support from peers [10]. Adjusting to the changes associated with the secondary school transition can be challenging. Unsuccessful negotiation may set some students on a trajectory of diminishing returns, not only in the short-term [11], [12], but also years thereafter [2].

### Effects of the secondary school transition on typically developing students' academic competence (AC) and mental health functioning (MHF)

Current evidence on the effects of the secondary school transition on AC (also referred to as academic performance or academic functioning) and MHF in typically developing students is mixed. Some studies suggest mean AC scores significantly decline after an initial settling-in period [3], [13]–[15]. Not every student experiences changes to the same extent, or even in the same direction [16], [17]. For example, less academically able students have been shown to have poorer adjustment to new school regimes [1], [18]. When compared to girls, boys have been shown to be more negatively affected by self-consciousness about AC, leading to declines in self-esteem and problems with adjustment subsequent to the transition [12]. The observed variability in AC across the transition has been attributed to various reasons including: study design and measurement issues (i.e., type of study, timing of data collection, stability and specificity of measurement tools used); social reference group variance; structural and philosophical differences between schools; and differences due to gender-role identification and personality [3], [15], [16], [18]–[23]. The role that the transition itself plays in the academic attainment dip is unclear, since a causal link between the transition and subsequent attainment has yet to be established [3].

Variability in student MHF is conspicuous within the transition literature. For example in one Australian study (restricted to two primary schools and one high school), the majority (55%) of students reported stable psychological health; 20% had better functioning, and 25% reported decreased psychological functioning through the transition [24]. Variability in MHF has also been reported in the US setting, where the middle school structure is common [25]–[27]. For example, Chung and colleagues [26] found students' MHF (from years 5 to 6) followed three trajectories (average start to high; low to moderately high; consistently high). Those with worse MHF prior to transition tended to have more adaptive difficulties after the transition when compared to their peers. Other studies report students with certain mental health conditions to be at a greater disadvantage across this transition. For example, studies suggest victimization is strongly related to depression, and weakly related to anxiety [28], while others suggest students with problem behaviours (disruptive or aggressive) have greater problems adjusting to junior high school [1], [29]. Gender differences in MHF have been documented. Girls report more internalising [30] and anxiety problems [31], while boys appear to exhibit more externalising problems [24], [32]. Overall, MHF across the primary-secondary transition varies widely amongst typically developing students. While some view the transition as demanding, others thrive on the challenges that it creates [33]. Therefore, considering a student cohort to be homogenous could be misleading.

### Limited focus on the impact of primary-secondary school transition on AC and MHF of students with disabilities

Few studies have considered the impact of transition to secondary school on AC and MHF of students with disabilities [34]. Students with learning disabilities have been reported to experience reductions in AC [35], while their typically developing counterparts show increased scores. Information on MHF of students with disabilities is variable, and depends on the construct used to define mental health. For example, when defined in terms of self-esteem, studies reported those with special educational needs (SEN) [36] and specific learning difficulties [35] to be at no greater risk than their peers. Students with special educational needs have, however, been reported to be at higher risk of bullying in secondary school (with 37% out of 110 reporting to be bullied when compared to 25% of their peers without SEN) [36]. In another study [37], while teachers reported students with specific learning difficulties to have significantly more internalising and externalising difficulties than their peers; students self-reported to have significantly fewer problems. Thus, measurement issues appear to confound the accuracy of self-reported mental health data for the disability subgroup [34].

### Effect of school contextual variables on AC and MHF

Although not explicit to the primary-secondary transition, there is inconclusive evidence on the influence of school contextual factors on AC and MHF. Various factors such as school size; sector; organisational system; and school socio-economic status (SES), which is based on the post-code of the school, have been implicated [38]–[44]. The contribution of school contextual factors to post-transition functioning in a mainstream Australian sample remains unexplored.

### Australian studies on the primary-secondary school transition

Case studies and literature reviews dominate the Australian literature on primary-secondary school transition [23], [45]–[52]. The available deductive studies are constrained by small sample size, design (i.e., convenience sampling) or scope (i.e., predominantly focus on mental health, bullying, or the changes in the school environment) issues that limit the generalisation of their findings [24], [33], [50], [53], [54]. With too few schools involved in transition research in Australia, exploration of ‘school effects’ on student AC and MHF across this transition is difficult (Fitz-Gibbon 1996; Smyth 1999). Similarly, for students with disabilities there are no Australian or international studies that have considered the impact of secondary school transition on perceived AC and MHF, despite inclusion of these students in the regular school system for decades [34]. The limited number and scope of studies precludes speculation on whether this subgroup may be additionally disadvantaged across the transition, even though they are at lower baseline level to their typically developing counterparts before the transition.

## Methods

### Aims and objectives

The overall aim of this study was to explore and compare perceived AC and MHF of students with and without disability, six months before and six months after transition to secondary school. The objectives were:

- determine the unique and combined effects of personal background factors (i.e., gender, disability and SES) on academic competence and mental health functioning before and after transition to secondary school;
- examine the added contribution of school contextual factors (i.e., sector, organisational structure, mean-school SES) on academic competence and mental health functioning before and after transition to secondary school, after accounting for personal background factors;
- determine the contribution of personal background factors and school contextual factors on change in academic competence and mental health functioning across the transition.

The current study is part of a larger study on the factors associated with student academic, social-emotional and participatory adjustment across the primary-secondary school transition [55].

### Design

A cohort study using a prospective, longitudinal design with two data collection points was used [Time 1 (T1) and Time 2 (T2)]. Informed written consent was obtained from school principals, parents, teachers, and written assent was obtained from students to participate in this study. In situations where the student declined to participate, even with parental consent, they were not included. All participants were made aware that they were not obliged to participate in the study, and were free to withdraw from the study at any time without justification or prejudice. At all stages, the study conformed to the National Health and Medical Research Council Ethics Guidelines [56]. Full ethics approval was obtained from Curtin University Health Research Ethics Committee, in Western Australia (WA) (Reference number HR 194/2005).

### Recruitment

The following inclusion criteria were applied to recruit students into the study:

1. Attending a regular school in the educational districts of metropolitan Perth or other major city centres of WA; and
2. Enrolled in the final year of primary school in WA (class 6 or 7) in the academic years commencing January 2006 or 2007, and due to transition to either middle or secondary school in January 2007 or 2008. Further details on the schooling system in WA are presented in Appendix A.

Students were categorised as having a disability if they were reported to have medical diagnosis or a disability or a chronic ill health condition, and were identified by their parent(s)/care-giver(s) to be attending a regular class for the majority of their weekly schooling hours (over 80% of the school hours per week), with support provided as required. Thus, a broad definition was used to define disability which entailed any addition medical health condition that had the potential of impact on an individual's daily functioning.

Several recruitment strategies were used to maximize reach and representativeness:

1. A pre-paid package (containing poster, letter of invitation, and school sector endorsement letter) was mailed out to principals from 250 primary schools listed on the Department of Education and Training, WA website. Schools listed in the Canning, Fremantle-Peel, Swan, and West Coast educational districts of Perth and major centres of Albany, Bunbury, Mid-West, Midlands, and Esperance educational districts of WA were approached.
2. A structured procedure was followed; with principal, teacher, parent and student consent obtained in that order.
3. A poster and a letter of invitation were circulated to the Disability Services Commission (DSC), the chief government body offering services to school-children with disability in WA. The DSC also posted a link to the study on its web page.
4. A pre-paid package (containing poster, letter of invitation, school sector and DSC endorsement letters) was circulated via known service providers, consumer groups, support groups, families of students with a disability via individual providers, and to any individuals who expressed interest in the study. In order to over sample the disability subgroup, additional snowball sampling occurred via participants forwarding information to friends and family.

T1 data collection was timed to ensure that parents had a definitive letter of acceptance from the secondary school, so that the identified secondary schools could be contacted at the commencement of the following academic year. The T1 parent questionnaire requested parents to list the name of the secondary school they planned to send their child to, for follow-up purposes. Follow-up of participants was carried out using the above mentioned recruitment procedure. T2 data collection commenced 6-months after the transition (Terms 3 and 4), after students had passed through the short-lived variability in functioning due to the transition, and had time to experience the new environments which either supported or hindered their transition.

### Data collection

Data were collected via questionnaires, primarily paper and pencil format. T1 data collection commenced in the second semester (Terms 3 and 4) of the final year in primary school (class 6 or 7) in the academic years commencing January 2006 or 2007. At T1, data from students, a parent (or primary caregiver) and the main class teacher were collected. To ensure consistency of administration, all questionnaires were administered on site by the first author or research assistants. Administration guidelines were developed to minimize administration bias. Student questionnaires were designed to be completed within one sitting during their regularly scheduled class time (35–40 minutes). On completion of this questionnaire, students returned it to staff and were given the pre-coded parent questionnaire in a reply-paid envelope, to take home. In cases where students were absent on the date of data collection, parent and student questionnaire packages (questionnaires and administration guidelines) were mailed-out to their residence. At T1, data from 395 students from 75 primary schools were collected. There were no more than 30 (11.3%) absent students across the schools sampled, across both academic years.

Routine follow-up protocol for parent/student/teacher questionnaires included: phone call to residence within two weeks; reminder mail if questionnaires were not returned within four weeks; and at least two fortnightly reminder phone calls.

T2 questionnaire administration commenced 6-months after the transition to secondary school. Administration was undertaken in the usual class times. Given that this was the second exposure to the survey, a decision was made to mail out 40% of the parent and student questionnaires to the students' residence, with the administration guideline and reply-paid envelope enclosed in the package. At T2, data from students and the same parent (primary caregiver) were collected. A student attrition rate of 32.7% resulted in a T2 sample of 266 participants from 152 secondary schools

### Power calculation

For the purpose of sample size estimation, it was assumed that there would be approximately 10 independent variables in the final regression model (for AC or MHF). In order to have power of .90 (*β* = 0.1) and with *α*-value of .05 (type I error), a sample size of 215 would be required to detect a small to moderate effect size of 0.1 (Sample Size Program: PASS) [57]. With an *α*-value of .05 and a β of .2, any between group comparisons with the smallest of groups, viz.: the 69 children with disabilities, allowed for a Cohen's *d* of .47 or larger to be detected.

### Data collection instruments

#### AC

Items from the scholastic competence domain of the Self-Perception Profile for Adolescents (SPPA) were used to measure student's perception of their AC (Harter, 1988). The SPPA has comparable internal consistency in populations of students with learning disability (α = 0.89), and behavioural disorders (α = 0.85) [58]. Considerate convergent, discriminant, and construct validity of the academic competence scale in an equivalent US and Australian sample has been substantiated [59]–[61]. Higher scores indicate better perceived AC.

#### MHF: The Strengths and Difficulties Questionnaire (SDQ)

The parent version of the Strengths and Difficulties Questionnaire (SDQ) was used to assess students' overall MHF [62]. The overall scores were computed by summing hyperactivity, emotional symptoms, conduct problems and peer problems subscales [62]. Moderate to high internal consistency values have been reported (α = 0.70–0.80) [63]. Empirical studies supported the tool's discriminate and predictive validity [62], [65]. The SDQ score correlates strongly with the Child Behaviour Checklist [64] but is more sensitive in detecting hyperactivity, and equally effective in detecting internalising and externalising problems in children and adolescents [65]. Australian norms have been published for the SDQ [63]. Higher scores indicate lower MHF.

#### Family demographics and school contextual characteristics

Family demographics: Items were drawn from the Indicators of Social and Family Functioning Instrument Version-1 (ISAFF) [66] and Australian Bureau of Statistics (ABS, 2001) surveys. Parents reported details on the family demographic characteristics, residence post code, and child's disability. Information on the school sector, post code number of students enrolled in each school, and organisational structure at each school was obtained from Department of Education and Training, WA records. The school post code was used to calculate its socio-economic index (SEIFA Index), using the Commonwealth Department of Education, Employment, and Workplace Relations measure of relative socio-economic advantage and disadvantage [67]. In this study, the SEIFA decile was used as the measure of mean school-SES, with a lower decile number meaning that the school was located in an area that is relatively more disadvantaged than other areas.

### Data Management

Data were managed and analysed using Statistical Package for Social Sciences Version 20 and Statistical Analysis Software Version 9.2. Data from the 2006 and 2007 cohort were alike on all factors. Hence, for purposes of subsequent analyses, sample scores were combined. Skewness/kurtosis measures indicated reasonable symmetry. Only 1.8–2.5% of data were missing at scale levels. The estimation maximization algorithm and Little's chi-square statistic identified data to be missing completely at random, with the probability level set at 0.05 [68], [69]. Standard guidelines recommended by the SDQ developers were followed to replace missing values and sensitivity checks were undertaken to substantiate the validity of data substitution techniques employed. Dummy variables were created to represent the categorical personal background and school contextual factors (i.e., independent variables) incorporated into the regression models [68].

### Analyses

The General Linear Model (GLM) procedure was used to address the study's objectives. The model was first tested with all personal background factors (i.e., gender, disability and SES) and their interactions. Since none of the interactions were statistically significant, they were removed from the model. The most parsimonious models including personal background and school contextual factors for each outcome at T1 and T2 are presented. The results from the model include the R^2^ or the proportion of variance in the outcome variable that could be explained by each personal background factor; the unstandardized regression coefficients (B) and their standard errors (SE), and the Least-Square (LS) means (or estimated population marginal means), which are within-group means appropriately adjusted for the other effects in the model [70].

## Results

At T1, data from 395 students from 75 primary schools were collected. Mean age of the students at T1 was 11.89 years (SD = 0.45 years, median = 12 years). A student attrition rate of 32.7% resulted in a T2 sample of 266 participants from 152 secondary schools. Chi-square and paired sample *t*-tests demonstrated that the participants who continued to be involved in the study at T2 did not differ from those who discontinued involvement, on gender, disability, SES-level, AC and MHF. The current paper presents data of the 266 students that answered questionnaires at T1 and T2. Access to the complete data can be obtained by contacting the first author.

Tables 1 and 2 give an overview of the key demographic characteristics of the student sample. The majority of the students in the disability subgroup had asthma, auditory disability, or a learning disability. Seventy six percent (n = 203) of students were from two-parent (original or biological) families, 11% (*n* = 29) were from the blended/extended/combination families, while the remaining 12.8% (n = 34) were from single-parent households. English was the main language spoken in 95.5% households (n = 252). Mothers of 23% (n = 60) of the sample did not have a post-school qualification. Of those who had a post school qualification, 5% (n = 13) completed a trade/apprenticeship course, 31.5% (n = 82) completed a vocational education and training certificate from college or Training and Further Education, 20% (n = 52) had a bachelor's degree, 20.4% (n = 53) had a post graduate degree. Eighty-two percent of the mothers (n = 218) were in paid employment, and 53.5% (n = 110) of the working mothers held professional/managerial employment titles. The remaining held clerical/administrative, technical, or sales positions. T2 data were collected after 12 months. The mean age of students at T2 was 12.9 years (SD = 0.57 years, median = 13 years).

**Table 1:** Student demographic characteristics at T1: Gender, health status, and household SES-level.

Characteristics	T1	
	N = 266	%
**Gender**		
Boy (Mean age 11.98 years, *SD* = 0.44 years)	124	46.6
Girl (Mean age 11.77 years, *SD* = 0.46 years)	142	53.4
**Health status**		
No Disability (Mean age 11.84years, *SD* = 0.41 years)	197	74.1
Disability (Mean age 11.96 years, *SD* = 0.58 years)	69	25.9
**Household SES-level ** [66], [122]		
$1–599/per week (low-SES level)	23	8.7
$600–1,999/per week (mid-SES level)	154	58.3
$2,000 +/per week (high-SES level)	87	33.0

**Table 2:** Types of disabilities involved in the student sample.

Disability type	n	%
Asthma	13	18.8
Auditory disability	11	15.9
LD	8	11.6
CP	6	8.7
ADHD	6	8.7
Asperger	5	7.2
Visual disability	5	7.2
ADD	4	5.8
Juvenile diabetes	2	2.9
Osteogen imperfecta	2	2.9
ASD	1	1.4
Brachial	1	1.4
Diabetes	1	1.4
Enuresis	1	1.4
Epilepsy	1	1.4
Haemophilia	1	1.4
Hypothyroidism	1	1.4
Total	69	100

Of the 250 primary schools invited to participate in the study, 175 declined, resulting in a non-participation rate of 70%. Only ten (14.9% of 67) students with disability were sourced from outside the main school recruitment (through DSC and the snowball). Details on the school characteristics of the 266 students surveyed at T1 and T2 are presented in Tables 3–5.

**Table 3:** School characteristics at T1 and T2. The same 266 subjects were surveyed at both time points.

	T1	T2
**School Sector**		
Catholic	77 (29.0%)	81 (40.5%)
Government	125 (47.0%)	79 (29.7%)
Independent private	64 (24.0%)	106 (39.9%)
**School organisational structure**		
Primary school (K-7)	209 (78.6%)	
Secondary school (Y8-10/12)		173 (65.0%)
K-12 without middle school	33 (12.4%)	52 (19.6%)
K-12 with middle school	24 (9.0%)	41 (15.4%)
**Mean school SES (indexed by SEIFA** 1 **decile)**		
1–6	45 (16.9%)	37 (13.9%)
7–8	47 (17.7%)	51 (19.2%)
9	117 (44.0%)	112 (42.1%)
10	57 (21.4%)	66 (24.8%)
**Primary school size based on total number of students**		
small = <375	67 (25.2%)	
mid-range = 375–975	141 (53.0%)	
large = >975	58 (21.8%)	
**Secondary school size based on total number of students**		
small = <700		67 (25.2%)
mid-range = 700–1250		120 (45.1%)
large = >1250		79 (29.7%)
**Year of transition**		
Year 6	26 (9.8%)	
Year 7	240 (90.2%)	
**Same secondary school as primary**		
No		211 (79.3%)
Yes		55 (20.7%)

1 The SEIFA decile was used as the measure of mean school-SES, with a lower decile number meaning that the school was located in area that is relatively more disadvantaged than other areas.

**Table 4:** Change in school sector of the total sample across the primary-secondary transition (N = 266).

T1 School sector		T2 School sector			
	Sector	Government	Catholic	Independent/Private	Sum (%)
	Government	75 (60.0%)	14 (11.2%)	36 (28.8%)	125 (100)
	Catholic	2 (2.6%)	66 (85.7%)	9 (11.7%)	77 (100)
	Independent/Private	2 (3.1%)	1 (1.6%)	61 (95.3%)	64 (100)

Numbers in each cell show the number of students and percentage of the school sector to which they belong.

**Table 5:** Change in school sector of the disability subgroups across the primary-secondary transition (n = 69).

T1 School sector		T2 School sector			
	Sector	Government	Catholic	Independent/Private	Sum (%)
	Government	25 (69.4%)	2(5.6%)	9 (25%)	36 (100)
	Catholic	1 (5.9%)	14 (82.3%)	2 (11.8%)	17 (100)
	Independent/Private	0 (0.0%)	0 (0.0%)	16 (100%)	16 (100)

Numbers in each cell show the number of students and percentage of the school sector to which they belong.

At T1, 47% of the students (n = 125) were enrolled in the public schools, 29% (n = 77) in Catholic schools and the remaining 24% (n = 64) in independent/private schools. There was a movement out of government schools towards Catholic and Independent schools at T2, with 60% staying in the government sector while over 85% of students in other sectors at T1 remained in those sectors at T2. Almost 80% (n = 209) were in schools that followed the K7–K10/12 organisational system. The majority of the sample at both T1 and T2 (T1 = 53.0%, n = 141; T2 = 45.1%, n = 120) received their education from mid-range sized schools. Slightly more than 90% (n = 240) moved to secondary school at the completion of Year 7, and 79% (n = 211) moved to a secondary school which was not connected with their primary school. Kappa statistics was used to determine whether the agreement between school sector attendance at T1 and T2 exceeded chance levels [71]. As shown in Tables 4 and 5, a significant change in school sectors accessed by the total sample and sub-group with disability across the transition was noticed (Kappa coefficient = 0.64).

### Model predicting AC at T1

As shown in Table 6, personal background factors explained 14.2% of the variability in T1 AC scores. While the models included a term for gender, this appeared not to be significantly associated with either the AC scores at T1 or T2, or the change in AC scores over the transition.

**Table 6:** Personal background and school contextual factors associated with perceived AC at T1 and T2, and across the T1–T2 transition (higher value represents better outcomes).

	Variable	B (SE)	LS-Mean	p-value
	**Model predicting T1 AC**			
**Step 1: Personal factors**	**Gender**			
**R^2^ = 14.2%**				
	Male	0.09 (0.08)	2.74	0.2818
	Female		2.66	
	**Presence of disability**			
	Yes	−0.42 (0.09)	2.49	<0.0001
	No		2.91	
	**Household SES**			
	low-SES = <$599	−0.67 (0.17)	2.35	<0.0001
	mid-range SES = $600–$1999	−0.30 (0.09)	2.73	
	high-SES = $2000+		3.02	
**Step2: School contextual factors**	**T1 school sector**			
**R^2^ = 17.3%**				
	Catholic	−0.62 (0.23)	2.40	0.0236
	Government	−0.48 (0.22)	2.54	
	Independent private		3.02	
	**T1 School size**			
	small = <375	0.61 (0.23)	2.84	0.0215
	mid-range = 375–975	0.64 (0.24)	2.88	
	large = >975		2.23	
	**Model predicting T2 AC**			
**Step 1: Personal factors**	**Gender**			
**R^2^ = 5.1%**				
	Male	0.13 (0.08)	2.91	0.1021
	Female		2.78	
	**Presence of disability**			
	Yes	−0.18 (0.09	2.75	0.0495
	No		2.93	
	**Household SES**			
	low-SES = <$599	−0.39 (0.16)	2.65	0.0226
	mid-range SES = $600–$1999	−0.19 (0.09)	2.85	
	high-SES = $2000+		3.03	
**Step2: School contextual factors**	**T1 school sector**			
**R^2^ = 10.5%**				
	Catholic	0.39 (0.38)	2.98	0.0131
	Government	0.85 (0.39)	3.44	
	Independent private		2.59	
	**T1 School size**			
	small = <375	0.85 (0.38)	3.27	0.0514
	mid-range = 375–975	0.92 (0.38)	3.33	
	large = >975		2.42	
	**Model predicting change in AC from T1 to T2**			
**Step 1: Personal factors**	**Gender**			
**R^2^ = 5.2%**				
	Male	0.04 (0.07)	0.16	0.5483
	Female		0.12	
	**Presence of disability**			
	Yes	0.23 (0.08)	0.26	0.0046
	No		0.03	
	**Household SES**			
	low-SES = <$599	0.28 (0.14)	0.29	0.1168
	mid-range SES = $600–$1999	0.11 (0.08)	0.12	
	high-SES = $2000+		0.01	
**Step2: School contextual factors**	**T1 school structure**			
**R^2^ = 9.8%**				
	K-12 with middle school	−0.46	−0.28	0.0016
	K-12 without middle school	0.06	0.24	
	Primary school (K-7)		0.19	

Students with disability reported significantly lower AC than their typically developing counterparts. Household SES was linearly related to T1 AC; with those from higher SES households having the highest AC scores and those from socially disadvantaged households having the lowest scores.

After accounting for personal background variability, school contextual factors could explain an additional 3.1% of the variability in T1 AC. In ascending order, students from Catholic schools reported lowest AC, followed by government, and independent sector students. Students attending larger schools appeared to have lower AC scores than the other schools.

### Model predicting AC at T2

At T2, personal background factors accounted for only 5.1% of the variance in AC. The disability subgroup continued to report lower AC than their typically developing peers (*p* = .0495), but the magnitude of this difference was not as large as reported at T1. The linear relationship between household-SES and T2 AC continued (high-SES>mid-range SES>low-SES); but the strength of this relationship reduced significantly.

After accounting for personal background variability, T2 school contextual factors could not explain any additional variability in T2 AC scores. T1 school size and organisational type continued to account for 5.4% of the variability in T2 AC scores. Attending large schools at T1 was associated with lower prospective AC (i.e., T2 AC). Students from T1 schools that used the K-7 organisational structure reported the lowest AC scores.

### Model predicting change in AC over the primary-secondary transition

Personal background factors explained 5.2% of the change in AC across the transition. Students with a disability showed an improvement in AC compared with other students. Those students who attended K-12 schools with middle school system appeared to show a reduced AC score across the transition compared to students from other school structure types.

### Model predicting MHF at T1

At T1, personal background factors explained 21.4% of the variability in MHF (Table 7). Boys and students with a disability had lower scores than girls and students without disability respectively. An inverse relationship between MHF and household-SES was evident. In descending order, students from higher-SES households had better MHF than those from mid-range, followed by low-SES. T1 School contextual factors could not explain any additional variability in MHF than the above-mentioned personal background factors.

**Table 7:** Personal background and school contextual factors associated with MHF at T1 and T2, and across the T1–T2 transition (higher value represents worse outcomes).

	Variable	B (SE)	LS-Mean	p-value
	**Model predicting T1 MHF**			
**Step 1: Personal factors**	**Gender**			
**R^2^ = 21.4%**				
	Male	1.40 (0.61)	9.47	0.0229
	Female		8.06	
	**Presence of disability**			
	Yes	4.82 (0.70)	11.18	<0.0001
	No		6.35	
	**Household SES**			
	low-SES = <$599	3.82 (1.24)	10.96	0.0078
	mid-range SES = $600–$1999	1.06 (0.66)	8.20	
	high-SES = $2000+		7.14	
**Step2: School contextual factors**	**T1 school structure**			
**R^2^ = 22.8%**				
	K-12 with middle school	0.12 (1.15)	9.05	0.0855
	K-12 without middle school	−2.13 (0.97)	6.81	
	Primary school (K-7)		8.93	
	**Model predicting T2 MHF**			
**Step 1: Personal factors**	**Gender**			
**R^2^ = 20.1%**				
	Male	1.09 (0.59)	9.11	0.0656
	Female		8.02	
	**Presence of disability**			
	Yes	4.58 (0.67)	10.86	<0.0001
	No		6.27	
	**Household SES**			
	low-SES = <$599	2.74 (1.23)	9.91	0.0228
	mid-range SES = $600–$1999	1.45 (0.63)	8.62	
	high-SES = $2000+		7.17	
**Step2: School contextual factors: Step 2: No school variables contribute further**				
	**Model predicting change in MHF from T1 to T2**			
**Step 1: Personal factors**	**Gender**			
**R^2^ = 2.1%**				
	Male	−0.13 (0.46)	−0.43	0.7755
	Female		−0.30	
	**Presence of disability**			
	Yes	−0.32 (0.53)	−0.53	0.5375
	No		−0.20	
	**Household SES**			
	low-SES = <$599	−1.78 (0.97)	−1.61	0.1075
	mid-range SES = $600–$1999	0.18 (0.50)	0.35	
	high-SES = $2000+		0.17	
**Step2: School contextual factors**	**T1 school sector**			
**R^2^ = 7.0%**				
	Catholic	−1.87 (0.64)	−0.70	0.0017
	Government	−2.10 (0.60)	−0.92	
	Independent		1.18	

### Model predicting MHF at T2

At T2, personal background variability explained 20.1% of the variability in MHF. The difference in MHF between genders narrows at T2 to the point that it is not statistically significant. The students with a disability appeared to have significantly lower MHF scores at T2 (similar to T1). The inverse linear relationship between household SES and MHF persisted after the secondary school transition. Similar to the T1 model, school contextual factors could not explain any variability in MHF additional to the personal background factors discussed above.

### Model predicting change in MHF over the primary-secondary transition

Personal background factors accounted for 2.1% of the change MHF across the transition, with none of them demonstrating statistically significant associations. An unexpected finding was the prospective impact of T1 school sector on change in MHF over the transition (explaining 5.9% of the MHF change). Students who attended independent schools at T1 reported lower MHF at T2, while those who attended other school sectors showed small improvements in their MHF.

## Discussion

Mixed evidence exists on the effects of this school transition on student AC and MHF. Researchers generally agree that no given student cohort is homogeneous [12], [72]. By employing a prospective longitudinal design, the current study examined the contribution of personal background factors (i.e., gender, presence of disability and household SES) and school contextual factors (i.e., size, sector, organisational model, and mean-SES) on perceived AC and overall MHF across the primary-secondary transition.

### Personal factors and AC and MHF at different times across the transition

The current study found that personal background factors explained the majority of the variability in student AC and MHF, before and after the transition. A significant reduction in the contribution of personal background factors on AC subsequent to the transition, despite AC scores staying stable across time was unexpected. This finding suggests that factors other than gender, disability and household-SES influence AC at T2 [55]. In the case of MHF, the contribution of personal background factors remained broadly constant at both times, a finding consistent with previous evidence [73].

Students with a disability had lower AC than their typically developing peers. This finding was in line with a number of previous studies [35], [58], [74], [75]. The reduced AC in the disability subgroup could be explained by the negative social comparison processes (referred to as the Big-fish-little-pond effect, (BFLPE) [76]. According to the BFLPE hypothesis, a student's self-concept is negatively correlated with one's peers. Thus, a student's academic self-concept depends not only on the student's academic accomplishments but also the accomplishments of those in the school that the student attends. The consistently lower AC in the disability sub-group found in the current study highlights the need for schools to recognise and address this issue.

Of interest was an improvement in the disability subgroup's AC after the transition. This finding could suggest that there was a less obvious BFLPE in secondary school, or the timing of data collection which was 6-months after the transition to secondary school was not long enough for ability groupings among students to be obvious. Long term longitudinal studies that track students through the secondary years of school would be beneficial in understanding the effect of regular secondary school attendance on the disability subgroup's AC relative to their typical peers, especially in light of evidence suggesting poorer school completion rates and employment participation rates among youth and young adults with disability [77], [78].

The consistent poorer MHF in the disability subgroup found in the current study could be attributed to several factors including biological processes (e.g., deficits in cognition; language and communication, social skills); the effect of medication; the psychological burden associated with having a disability; or the associations between mental disorders and lifestyle risk factors [79]–[86]. Given the importance of positive mental health in itself and the detrimental impact of mental ill health on the individual and society over time [87]–[94], the current study's findings reinforce the importance of comprehensive, whole-of school, mental health prevention programs currently operational in Australian primary and secondary schools [95]–[97].

With regards to gender and AC, our study's findings support egalitarian theories of the reduced gender-stereotyped socialization over the past decade. This could be attributed to interventions and legislation aimed at increasing girls' motivation and participation in academic pursuits. Future research into whether egalitarian patterns hold in subject-specific academic domains (i.e., math, computers, sciences, history) could help target specific interventions for those most in need. In the case of MHF, a significant gender bias favouring girls was evident in primary school. This finding could be a function of poorer behaviour during the last term of the school year (i.e., an effect of timing of measurement). The improvement in overall MHF in boys after the transition and the absence of any significant gender association with MHF in secondary school is a positive finding. Our findings highlight the need for primary and secondary schools to be equally sensitive and responsive to the AC and MHF needs of boys and girls.

Consistent with earlier research [1], [15], the detrimental effects of social disadvantage on AC and MHF was evident. According to the Family Investment Model (FIM), higher SES households can afford to make significant capital investments in the development of their children, while more disadvantaged families are forced to invest in more immediate needs [98], [99]. Economic deprivation affects families' well-being through an increase in family stress, which in turn decreases ability to provide stability, adequate attention, supervision, and cognitive stimulation to children [100]. The absence of any cumulative disadvantage of household-SES on AC and MHF is optimistic. Furthermore, the reduced strength of the association between social disadvantage and AC and MHF post-transition, due to the improvement in the functioning of the lower-SES group could be attributed to several factors, which include: the transition trend noted in the study (i.e., increased enrolment in independent schools); or the effect of measurement (i.e., ceiling effect of the scales used, or the small sample size of the low-SES group making the detection of significant differences difficult due to power issues); or an indication that the transition to secondary school is beneficial to the MHF and AC of students from lower-SES subgroups. Nonetheless, this sub-group needs support more than their more affluent peers.

### School contextual factors and AC and MHF at different times across the transition

Across the board, school contextual factors explained very little, if any, of the variability in MHF, but more of the change in MHF over time. These findings concur with past findings on the small contribution of school factors on student MHF [101]–[104] and relatively larger contribution on AC [105], [106] and indicate that most school contextual factors provide similar experiences [107] or that school contextual factors are less important than personal background factors on student AC and MHF. Furthermore, no secondary school contextual characteristic (i.e., size, school sector, organisational model, mean-school SES or their interactions) influenced AC and MHF in secondary school. This means that individual student factors and primary school contextual factors are more important contributors of post-transition adjustment than concurrent secondary school contextual factors. Thus, there exists a greater responsibility on primary schools to ensure that the transition needs of the disadvantaged groups are satisfactorily met.

To date, the effect of school sector (private or public) on student outcomes is uncertain. Some findings suggest that that once student-household SES is considered in the analysis, the advantage of private schooling (independent schools) disappears or becomes minimal [38], [39]. Others suggest beneficial outcomes for those in private education [40], [108], [109]. In the current study, we found that even after accounting for personal background factors, attending an independent primary school was associated with higher concurrent AC but worse prospective MHF. The benefits of independent school attendance on AC could be attributed to the better resources, more functional and supportive school climate, or fewer discipline problems noted in these schools [40]. The lower AC found amongst those who attended Catholic schools is an unexpected finding which is contrary to past studies that highlight the benefits of Catholic school attendance in terms of a steady stream of funds that permits forward planning and budgeting, and institutional autonomy [40].

A trend was noticed for the whole student sample including the disability sub-group students to move out of government schools into independent or private schools for secondary education. This has been observed in previous Australian studies [41], [42]. Despite this transition trend, the absence of any significant contribution of school SES (indexed by the SEIFA score) on AC and MHF after adjustment for personal background factors validates the applicability of whole of school mental health models across school sectors, irrespective of social stratification. This finding is positive and suggests that for our current sample, individual-household SES was more important than the mean-school SES as far as AC and MHF were concerned. Given the relative skewness of the participating schools to higher deciles, it is likely that the detection of significant differences was difficult due to power issues. Caution ought to be exercised while interpreting these findings.

## Limitations

The sample of students was drawn from the Perth metropolitan area and major city centres across WA, and did not involve students from other rural and regional populations, or other major metropolitan cities in Australia. Despite extensive recruitment efforts, 70% of the schools declined to participate in the study, which may have introduced a possible bias. The study's cohort comprised 29% Catholic, 47% Government, and 24% Independent schools, which was different to the profile of all schools in Western Australia (15%, 72%, and 13% respectively) and may limit the generalizability of the findings.

The majority of the students in the disability subgroup had asthma, auditory disability, or a learning disability. The criteria for inclusion into the disability category could have resulted in the exclusion of students with more disability related physical, cognitive, social, and emotional restrictions [110]. Thus, the findings of the current study may underestimate the impact of more severe disabilities on school transition. Statistically, it is also likely that combining the reports of a heterogeneous disability subgroup, with less disability related limitations, may have reduced the sensitivity of the analyses [111]. Additionally, we did not account for the confounding effect of disability severity and comorbidity status on AC and MHF [112]. Replication of the study findings in students from other school settings, such as educational support units, separate schools that cater for students with severe disabilities or students who were home schooled and more severe disabilities is needed to extend generalizability.

In the current study, AC was evaluated by students only. Social desirability self-report bias may have exaggerated the relationship between the predictor variables and student perceived AC scores. Parents reported on their child's overall mental functioning using the SDQ, which tends to over emphasise externalising conduct features. Especially during the adolescent years, it is likely that children are more apt to have better insights into their own MHF than their parents. Additional research that involves multisource data from students, parents, teachers, and possibly clinical interviews and school records, is warranted to validate these findings [113].

Consistent with past studies [114], students from low-SES households were under-represented in our sample. Despite small numbers, the significant disadvantage found in this sub-group suggest that these students are greatly disadvantaged (i.e., the true effect size could be larger). We did not explicitly define the sub-group of individuals from Indigenous and Torres Strait communities due to ethical concerns. Further research is warranted to find out whether the findings of this study can be generalised to all sub-groups of the Australian population.

Also, the two-point longitudinal study design did not permit us study the longer-term effect of transition on AC and MHF. This is an area worthy of scrutiny.

## Conclusions

The current study is one of the few studies that investigated the effects of personal background and school contextual factors on AC and MHF across the primary-secondary transition, using a student sample with and without disabilities. Our findings highlight the existence of within-group variability in student AC and MHF and the responsibility on primary schools to ensure that the needs of disadvantaged groups are satisfactorily met, as these students continue to be disadvantaged after the transition to secondary school.

It is acknowledged that risks commonly accumulate and cluster across multiple contexts of development [115], [116]. Our findings highlight the need for detailed, multi-contextual assessment of personal background and school contextual factors that influence student AC and MHF across the primary-secondary school transition. Such studies are invaluable in guiding transition-specific interventions for all students in the regular school system.

## Appendix A: The schooling system in WA

Schooling in Western Australia (WA) is delivered under the State's Education Act (1999), the Curriculum Council Act (1997) and the Adelaide Declaration on National Goals for Schooling in the Twenty-first Century (MCEETYA, 2004). The concept of inclusion is firmly embedded within the WA Curriculum Framework [117], [118].

WA has government (public) school and non-government (private) school sectors. Government schools operate under the direct responsibility of the State Minister of Education and Training, and are represented by the Department of Education and Training (DET). The non-government sector is represented by the Catholic Education Office (CEO) and the Association of Independent Schools (AISWA). One-third of all students in Australia study in non-government schools, the majority of whom are from middle and upper socio-economic status (SES) background [119]. WA government schools are all co-educational. The privatised sector has co-educational and single-gender schools at primary and at secondary level.

Predominately, a three-stage educational structure consisting of pre-primary, primary, and secondary operates in most government and non-government schools. Schools organisational structures range from traditional primary-secondary school configurations (Kindergarten – Year 7, and Years 8–12), through separate structures within larger frameworks from Kindergarten - Year 12 (K-12), to specially designated middle schools (Year 6/7-Year 8 or 10/12) [120]. There are relatively few designated middle schools in WA when compared to the US and the rest of Australia [120]. During the time of data collection for this study, primary-secondary secondary school transition in WA occurred at the completion of Year seven (i.e., the year in which students turned 13). Post 2009, as part of a state-wide planning framework, a phased relocation of Year 7 students into the secondary settings is being undertaken on case-by-case [121].

Additionally, the models of inclusion for students with disabilities adopted in schools across WA vary widely with regard to student contact time in the regular classroom. In some inclusive instances, students with disabilities who are based in regular classrooms spend some time in specialised units or classes designed to cater to their needs. Students with a chronic illness also spend time out in hospital/home, or require assistance from nurses at school. The term regular schools in this paper is used to refer to a mainstreamed situation, in which students attend a regular class for almost 80% of the school hours per week, with support from specialised service providers offered as required.

## References

[1] Anderson LH, Jacobs J, Schramm S, Splittgerber F (2000) School transitions: Beginning of the end or a new beginning? International Journal of Educational Research 33.

[2] Seidman E, French S (2004) Developmental trajectories and ecological transitions: A two-step procedure to aid in choice of prevention and promotion interventions. Development and Psychopathology 16: 1141–1159. 15704831 10.1017/s0954579404040179

[3] West P, Sweeting J, Young R (2010) Transition matters: pupils' experiences of the primary–secondary school transition in the West of Scotland and consequences for well-being and attainment. Research Papers in Education 25: 21–50.

[4] Humphrey N, Ainscow M (2006) Transition Club: Facilitating Learning, Participation and Psychological Adjustment during the Transition to Secondary School. European Journal of Psychology of Education 21: 319–331.

[5] Hargreaves A (1986) Two cultures of schooling: The case of middle schools. London: Falmer Press.

[6] Juvonen J, Vi-Nhuan L, Kaganoff T, Augustine C, Constant L (2004) Focus on the wonder years: Challenges facing the American middle school. Santa Monica: Rand Corporation.

[7] Eccles JS, Midgley C, Wigfield A, Buchanan CM, Reuman D, et al (1993) Development during adolescence: The impact of stage-environment fit on adolescents' experiences in school and in families. American Psychologist 48: 90–101. 8442578 10.1037//0003-066x.48.2.90

[8] Erikson EH (1968) Identity:Youth and crisis. New York: Norton.

[9] Weiss RS (1973) Loneliness: The experience of emotional and social isolation. Cambridge, MA: MIT Press.

[10] Larson RW, Verma S (1999) How children and adolescents spend time across the world: Work, play, and developmental opportunities. Psychological Bulletin 125: 701–736. 10589300 10.1037/0033-2909.125.6.701

[11] Roeser RW, Strobel K, Quihuis G (2002) Studying early adolescents' academic motivation, social-emotional functioning, and engagement in learning: Variable and person-centered approaches. Anxiety, Stress and Coping 15: 345–368.

[12] Lord S, Eccles JS, McCarthy K (1994) Surviving the junior high school transition: Family processes and self-perceptions as protective risk factors. Journal of Early Adolescence 14: 162–199.

[13] Wigfield A, Eccles JS (1994) Children's Competence Beliefs, Achievement Values, and General Self-Esteem: Change Across Elementary and Middle School. Journal of Early Adolescence 14: 107–138.

[14] Watt HMG (2004) Development of adolescents' self perceptions, values and task perceptions according to gender and domain in 7th through 11th grade Australian students. Child Development 75: 1556–1574. 15369531 10.1111/j.1467-8624.2004.00757.x

[15] McGee CR, Ward J, Gibbons J, Harlow A (2003) Transition to secondary school: A literature review. A Report to the Ministry of Education. Hamilton, University of Waikato, New Zealand

[16] Block J, Robins RW (1993) A longitudinal study of consistency and change in self-esteem from early adolescence to early adulthood. Child Development 64: 909–923. 8339703 10.1111/j.1467-8624.1993.tb02951.x

[17] Harter S (1999) The construction of self: A developmental perspective. New York: Guilford.

[18] Galton M, Morrison I, Pell T (2000) Transfer and transition in English schools: reviewing the evidence. International Journal of Educational Research 33: 341–363.

[19] Marsh HW (1989) Age and sex effects in multiple dimensions of self-concept: Preadolescence to early adulthood. Journal of Educational Psychology 81: 417–430.

[20] Nottlemann E (1987) Competence and self-esteem during the transition from childhood to adolescence. Developmental Psychology 23: 441–450.

[21] Simmons R, Blyth D (1987) Moving into adolescence: The impact of pubertal change and school context. Hawthorn, NJ: Aldine de Gruyter.

[22] Eccles JS, Midgley C (1989) Stage-environment fit: Developmentally appropriate classrooms for young adolescents. In: Ames R, Ames C, editors. Research on motivation in education. New York: Academic Press. pp. 139–181.

[23] Luke A, Elkins J, Weir K, Land R, Carrington V, et al. (2003) Beyond the middle: A report about literacy and numeracy development of target group students in the middle years of schooling, Volume 1. In: Commonwealth Department of Education SaTatUoQ, editor.

[24] Wallis J, Barrett PM (1998) Adolescent adjustment and the transition into high school. Journal of Child and Family Studies 7: 43–58.

[25] Barber BK, Olsen JA (2004) Assessing the transitions to middle and high school. Journal of Adolesent Research 19: 3–30.

[26] Chung H, Elias M, Schneider K (1998) Patterns of individual adjustment change during middle school transition. The Journal of School Psychology 92: 20–25.

[27] Lohaus A, Elben C, Ball J, Klein-Hessling J (2004) School transition from elementary to secondary school: changes in psychological adjustment. Educational Psychology 24: 161–173.

[28] Hawker DSJ, Boulton MJ (2000) Twenty Years' Research on Peer Victimization and Psychosocial Maladjustment: A Meta-analytic Review of Cross-sectional Studies. Journal of Child Psychology and Psychiatry 41: 441–455. 10836674

[29] Berndt TJ, Mekos D (1995) Adolescents' perceptions of the stressful and desirable aspects the transition to junior high school. Journal of Research on Adolescence 5: 123–142.

[30] Anderman EM (2002) School effects on psychological outcomes during adolescence. Journal of Educational Psychology 94: 795–808.

[31] Benner AD, Graham S (2009) The transition to high school as a developmental process among multi-ethnic urban youth. Child Development 80: 356–376. 19466997 10.1111/j.1467-8624.2009.01265.x

[32] Roderick M (2003) What's happening to the boys? Early high school experiences and school outcomes among African American male adolescents in Chicago. Urban Education 38: 538–607.

[33] Bahr N, Pendergast D (2007) The millennial adolescent. Canberra: Australian Council for Educational Research.

[34] Hughes LA, Banks P, Terras MM (2013) Secondary school transition for children with special educational needs: a literature review. Support for Learning 28: 24–34.

[35] Forgan JW, Vaughn S (2000) Adolescents With and Without LD Make the Transition to Middle School. Journal of Learning Disabilities 33: 33–43. 15505954 10.1177/002221940003300107

[36] Evangelous M, Vangelous M, Taggart B, Sylva K, Melhuish E, et al. (2008) Effective Preschool, Primary and Secondary Education 3–14 Project (EPPSE 3–14): What Makes a Successful Transition from Primary to Secondary School ? Nottingham: DCSF Publications.

[37] Tur-Kaspa H (2002) The socioemotional adjustment of adolescents with LD in the kibbutz during high school transition periods. Journal of Learning Disabilities 35: 87–96. 15490902 10.1177/002221940203500107

[38] Organisation for Economic Co-operation and Development [OECD] (2003) School factors related to quality and equity: Results from PISA 2000. Paris: OECD.

[39] Gorard S (2006) The true impact of school diversity? In: Hewlett M, Pring R, Tulloch M, editors. Comprehensive education: evolution, achievement and new directions. Northampton: University of Northampton Press.

[40] Perry LB (2007) School composition and student outcomes: A review of emerging areas of research 2007; Fremantle, WA. Murdoch University

[41] Lamb S (2007) School reform and inequality in urban Australia: A case of residualising the poor. In: Teese R, Lamb S, Duru-Belat M, editors. Education and Inequality Dordrecht: Springer. pp. 1–38.

[42] Lamb S, Long M, Baldwin G (2004) Performance of the Australian education and training system. Melbourne: Centre for Post compulsory Education and Lifelong Learning, University of Melbourne.

[43] Lamb S, Walstab A, Teese R, Vickers M, Rumberger R (2004) Staying on at school: Improving student retention in Australia. Brisbane: Queensland Department of Education and the Arts.

[44] Bonell C, Parry W, Wells H, Jamal F, Fletcher A, et al (2012) The effects of the school environment on student health: A systematic review of multi-level studies. Health & Place 10.1016/j.healthplace.2012.12.001 23501377

[45] Edgar D (2001) The patchwork nation: Re-thinking government – re-building community. Pymble, N.S.W.: HarperCollins.

[46] Lo Bianco J, Freebody P (1997) Australian literacies: Informing national policies on literacy education. Melbourne: Language Australia.

[47] Luke A, Land R, Christie P, Kolatsis A, Noblett G (2002) Standard Australian english and languages for Queensland Aboriginal and Torres Strait Islander students. Brisbane: Indigenous Education Consultative Body.

[48] Hanewald R (2013) Transition Between Primary and Secondary School: Why it is Important and How it can be Supported. Australian Journal of Teacher Education 38.

[49] Howard S, Johnson B (2004)Transition from primary to secondary school: Possibilities and paradoxes.

[50] Kirkpatrick D (1993) Student perceptions of the transition from primary to secondary school. Australian Association for Research in Education Conference. Geelong.

[51] Kirkpatrick D (1997) Making the Change. The transition from primary to secondary school. Education Australia 36: 17–19.

[52] Marston J (2008) Perceptions of students and parents involved in primary to secondary school tranistion programs. Australian Association for Research in Education. Brisbane: Australian Association for Research in Education.

[53] Chadbourne R (2001) Middle schooling for the middle years. What might the jury be considering?. Southbank, Victoria: Australian Education Union.

[54] Carrington V, Pendergast D, Bahr N, Kapitzke C, Mayer D, et al (2002) Education futures: Transforming teacher education (Re-framing teacher education for the middle years). Proceedings of the 2001 National Biennial Conference of the Australian Curriculum Studies Association

[55] Vaz S (2010) Factors affecting student adjustment as they transition from primary to secondary school: A longitudinal investigation. Perth: Curtin University.

[56] National Health and Medical Research Centre [NHMRC] (2005) Human research ethics handbook: A research law collection.

[57] NCSS (1996) PASS 6.0 user's guide. Kaysville, UT: NCSS.

[58] Harter S, Whitesell N, Junkin L (1998) Similarities and differences in domain-specific and global self-evaluations of learning disabled, behaviorally disordered, and normally achieving adolescents. American Educational Research Journal 35: 653–680.

[59] Zubrick S, Silburn SR, Garton A (1993) Field instrument development for the Western Australian child health study. Perth, WA: Western Australian Research Institute for Child Health.

[60] Passmore A (1998) The relationship between leisure and mental health in adolescents. Perth University of Western Australia.

[61] Harter S (1982) The perceived competence scale for children. Child Development 53: 87–97. 6525886

[62] Goodman R (1997) The strengths and difficulties questionnaire: A research note. Journal of Child Psychology and Psychiatry 38: 581–586. 9255702 10.1111/j.1469-7610.1997.tb01545.x

[63] Mellor D (2005) Normative data for the strengths and difficulties questionnaire in Australia. Australian Psychologist 40: 215–222.

[64] Achenbach TM (1991) Manual for the youth self-report and 1991 profile. Burlington, VT: University of Vermont Department of Psychiatry

[65] Goodman R, Scott S (1999) Comparing the Strengths and Difficulties Questionnaire and the Child Behaviour Checklist: Is small beautiful? Journal of Abnormal Child Psychology 1. 10.1023/a:1022658222914 10197403

[66] Zubrick S, Williams A, Silburn SR, Vimpani G (2000) Indicators of social and family functioning. Canberra: Commonwealth of Australia.

[67] Australian Bureau of Statistics [ABS] (2011) 2033.0.55.001 - Census of Population and Housing: Socio-Economic Indexes for Areas (SEIFA). Canberra: ABS.

[68] Meyers LS, Gamst G, Guarino AJ (2006) Applied multivariate research: Design and implication. CA: Sage Publications, Inc.

[69] Tabachnick B, Fidell L (2007) Using multivariate statistics. Boston, MA: Pearson Education Inc. and Allyn & Bacon.

[70] Tabachnick B, Fidell L (2001) Using multivariate statistics Boston, MA: Allyn and Bacon.

[71] Cohen JM (1960) A coefficient of agreement for nominal scales. Educational and Psychological Measurement 20: 37–46.

[72] Bahr N (2005) The middle years learner. In: Pendergast D, Bahr N, editors. Teaching middle years: Rethinking curriculum, pedagogy and assessment Crows Nest, NSW: National Middle School Association pp. 49–64.

[73] Davis C, Martin G, Kosky R, O'Hanlon A (2000) Early Intervention in the Mental Health of Young People: A Literature Review. Monograph published by The Australian Early Intervention Network for Mental Health in Young People.

[74] Snowling M, Muter V, Carroll J (2007) Outcomes in adolescence of children at family-risk of dyslexia. Journal of Child Psychology & Psychiatry 48: 609–618. 17537077 10.1111/j.1469-7610.2006.01725.x

[75] Zeleke S (2004) Self-concepts of students with learning disabilities and their normally achieving peers: a review. European Journal of Special Needs Education 19: 145–170.

[76] Marsh HW, Hau KT (2004) Big fish, little pond effect. On academic self-concept. Self Concept Research, Driving International Research Agendas. Manly.

[77] Howlin P, Moss P (2012) Adults with autism spectrum disorders. The Canadian Journal of Psychiatry 57: 275–283. 22546059 10.1177/070674371205700502

[78] AVECO (2012) Youth unemployment: The crisis we cannot afford. London: AVECO.

[79] Haager D, Vaughn S (1995) Parent, teacher, and self-reports of the social competence of students with learning disabilities. Journal of Learning Disabilities 28: 205–215. 7738433 10.1177/002221949502800403

[80] Newman L (2004) Family involvement in the educational development of youth with disabilities: A special topic report of findings from the national longitudinal transition study-2 (NLTS-2). Menlo Park, CA: SRI International.

[81] Prince M, Patel V, Saxena S, Maj M, Maselko J, et al (2007) No health without mental health. The Lancet 370: 859–877. 10.1016/S0140-6736(07)61238-0 17804063

[82] Lucas RE (2007) Long-term disability is associated with lasting changes in subjective well-being: evidence from two nationally representative longitudinal studies. J Person Soc Psychol 92: 717–730. 10.1037/0022-3514.92.4.717 17469954

[83] Emerson E, Honey A, Llewellyn G, Madden R (2009) The Well-being of Australian Adolescents and Young Adults with Self-reported Long-term Health Conditions, Impairments or Disabilities: 2001 and 2006. Australian Journal of Social Issues 44: 39–54.

[84] Gallo LC, Matthews KA (2003) Understanding the association between socioeconomic status and physical health: do negative emotions play a role? Psychol Bull 129: 10–51. 12555793 10.1037/0033-2909.129.1.10

[85] Sawyer MG, Arney FM, Baghurst PA, Clark JJ, Graetz BW, et al. (2000) The mental health of young people in Australia: Child and adolescent component of the National Survey of Mental Health and Wellbeing. Canberra, Australia: Department of Health and Aged Care, Mental Health and Special Programs Branch.

[86] Wight RG, Botticello AL, Aneshensel CS (2006) Socioeconomic context, social support, and adolescent mental health: A multilevel investigation. Journal of Youth and Adolescence 35: 115–126.

[87] Zubrick S, Silburn SR, Gurrin L, Teoh H, Shepherd C, et al. (1997) The Western Australian Child Health Survey: Education, health and competence. Perth, WA: Australian Bureau of Statistics and the Institute for Child Health Research.

[88] Zubrick S, Silburn SR, Burton P, Blair EM (2000) Mental health disorders in children and young people: Scope, cause and prevention. Australian and New Zealand Journal of Psychiatry 34: 570–578. 10954387 10.1080/j.1440-1614.2000.00703.x

[89] Rutter M (1995) Relationships between mental disorders in childhood and adulthood. Acta Psychiatrica Scandinavica 91: 73–85. 7778474 10.1111/j.1600-0447.1995.tb09745.x

[90] Petticrew M, Chisholm D, Thomson H, Jane-LLlopis E (2005) Generating evidence on determinants, effectiveness and cost effectiveness. In: Herrman I, Saxena H, Moodie R, editors. Promoting Mental Health: Concepts, Emerging Evidence, Practice. Geneva: World Health Organisation.

[91] Geller B, Zimerman B, Williams M, Bolhofner K, Craney JL (2001) Bipolar disorder at prospective follow-up of adults who had prepubertal major depressive disorder. American Journal of Psychiatry 158: 125–127. 11136645 10.1176/appi.ajp.158.1.125

[92] Geller B, Zimerman B, Williams M, Bolhofner K, Craney JL (2001) Adult Psychosocial Outcome of Prepubertal Major Depressive Disorder. Journal of the American Academy of Child and Adolescent Psychiatry 40: 673–677. 11392345 10.1097/00004583-200106000-00012

[93] Orvaschel H, Lewinsohn PM, Seeley JR (1995) Continuity of Psychopathology in a Community Sample of Adolescents. Journal of the American Academy of Child and Adolescent Psychiatry 34: 1525–1535. 8543521 10.1097/00004583-199511000-00020

[94] Cicchetti D, Rogosch FA (1999) Psychopathology as Risk for Adolescent Substance Use Disorders: A Developmental Psychopathology Perspective. Journal of Clinical Child Psychology 28: 355–365. 10446685 10.1207/S15374424jccp280308

[95] Roberts C, Ballantyne F, Van der Klift P (2002) Aussie Optimism: Social life skills program. Teacher resource. Perth, WA: Curtin University of Technology.

[96] Cross D, Erceg E (2002) Friendly schools and families. Perth: Edith Cowan University.

[97] World Health Organisation [WHO] (1996) Promoting health through schools - The World Health Organisation's global school health initiative. Geneva: World Health Organisation.

[98] Becker GS, Thomes N (1986) Human capital and the rise and fall of families. Journal of Labor Economics 4: 1–139. 12146356 10.1086/298118

[99] Bradley RH, Corwyn RF (2002) Socio-economic status and child development. Annual Review of Psychology 53: 371–399. 10.1146/annurev.psych.53.100901.135233 11752490

[100] Hauser RM, Brown BV, Prosser WR (1997) Indicators of children's wellbeing New York Russell Sage Foundation

[101] Saab H, Klinger D (2010) School differences in adolescent health and wellbeing: Findings from the Canadian Health Behaviour in School-aged Children Study. Social Science & Medicine 70: 850–858. 20089340 10.1016/j.socscimed.2009.11.012

[102] Andersson H, Bjørngaard J, Kaspersen S, Wang CA, Skre I, et al (2010) The effects of individual factors and school environment on mental health and prejudiced attitudes among Norwegian adolescents. Social Psychiatry and Psychiatric Epidemiology 45: 569–577. 19629360 10.1007/s00127-009-0099-0

[103] Roeger L, Allison S, Martin G, Dadds V, Keeves J (2001) Adolescent depressive symptomatology. Australian Journal of Psychology 53: 134–139.

[104] Konu AI, Lintonen TP, Autio VJ (2002) Evaluation of Well-Being in Schools - A Multilevel Analysis of General Subjective Well-Being. School Effectiveness and School Improvement 13: 187–200.

[105] Hattie J (1999) Influences on student learning. University of Auckland

[106] Hattie J (2003) Teachers make a difference: What is the research evidence? Council for Educational Research Annual Conference on Building Teacher Quality. Melbourne.

[107] Rutter M, Maughan B (2002) School Effectiveness Findings 1979–2002. Journal of School Psychology 40: 451–475.

[108] Waters SK, Cross D, Shaw T (2010) Does the nature of schools matter? An exploration of selected school ecology factors on adolescent perceptions of school connectedness. British Journal of Educational Psychology 80: 381–402. 20109276 10.1348/000709909X484479

[109] Waters SK, Cross D, Shaw T (2010) How important are school and interpersonal student characteristics in determining later adolescent school connectedness, by school sector? Australian Journal of Education 54: 6.

[110] Bell M, Dempsey I (2001) Enrolment and placement practices for students with special needs in NSW government schools: A critical analysis of current policy. Special Education Perspectives 10: 3–14.

[111] Portney LG, Watkins MP (2000) Foundations of clinical research: Applications to practice. Upper Saddle River, New Jersey: Prentice- Hall.

[112] Yeo M, Sawyer S (2005) Chronic illness and disability. British Medical Journal 330: 721–723. 15790645 10.1136/bmj.330.7493.721 PMC555640

[113] Stone LL, Otten R, Engels RC, Vermulst AA, Janssens JM (2010) Psychometric properties of the parent and teacher versions of the strengths and difficulties questionnaire for 4- to 12-year-olds: a review. Clinical child and family psychology review 13: 254–274. 20589428 10.1007/s10567-010-0071-2 PMC2919684

[114] Kipke RC (2008) Culture in rvaluation #6: Low socio-economic status populations in California. UC Davis: Tobacco Control Evaluation Center.

[115] Gutman LM, Sameroff AJ, Cole R (2003) Academic growth curve trajectories from 1st grade to 12th grade: effects of multiple social risk factors and preschool child factors. Dev Psychol 39: 777–790. 12859129 10.1037/0012-1649.39.4.777

[116] Jimerson S, Egeland B, Sroufe LA, Carlson B (2000) A Prospective Longitudinal Study of High School Dropouts Examining Multiple Predictors Across Development. Journal of School Psychology 38: 525–549.

[117] Council Curriculum (1998) Curriculum framework for kindergarten to year 12 education in Western Australia. Perth. Western Australia.

[118] Department of Education and Training [DET] (2004) Pathways to the Future: A Report of the Review of Educational Services for Students with Disabilities in Government Schools. East Perth, W.A.

[119] Ryan C, Watson L (2004) The drift to private schools in Australia: Understanding its features (No. Discussion Paper No. 479). Australian National University.

[120] Council of Government School Organisations [COGSA] (2005) Middle schooling concepts and approaches: A council of government school organisations' discussion paper. Northern Territory: The Northern Territory Council of Government School Organisations.

[121] Department of Education and Training [DET] (2007) The future placement of year 7 students in WA public schools: A study. Perth Government of Western Australia.

[122] Australian Bureau of Statistics [ABS] (2001) ABS 2001 Census Dictionary (Cat. No. 2901.0).

